# Neutrophil-to-Lymphocyte Ratio as a Prognostic Biomarker for Long-Term Survival in Older Adults at a Mental Health Care Center: A Historical Cohort Analysis

**DOI:** 10.3390/jcm14072509

**Published:** 2025-04-07

**Authors:** Piotr Paweł Chmielewski, Bartłomiej Strzelec, Paul Mozdziak, Bartosz Kempisty

**Affiliations:** 1 Division of Anatomy, Department of Human Morphology and Embryology, Faculty of Medicine, Wroclaw Medical University, 6a Chalubinskiego Street, 50-368 Wroclaw, Poland; bartosz.kempisty@umw.edu.pl; 2 2nd Department of General Surgery and Surgical Oncology, Medical University Hospital, 50-345 Wroclaw, Poland; bstrzelec94@interia.pl; 3 Prestige Department of Poultry Science, College of Agriculture and Life Sciences, North Carolina State University, Raleigh, NC 27695-7608, USA; pemozdzi@ncsu.edu; 4 Department of Veterinary Surgery, Institute of Veterinary Medicine, Nicolaus Copernicus University, 87-100 Toruń, Poland; 5 Center of Assisted Reproduction, Department of Obstetrics and Gynecology, University Hospital and Masaryk University, 625 00 Brno, Czech Republic

**Keywords:** aging, chronic inflammation, inflammation, longevity, longevity biomarkers, neutrophil-to-lymphocyte ratio, immunosenescence, senescence, survival, systemic inflammation

## Abstract

**Background/Objectives**: Identifying reliable biomarkers for healthy aging and longevity is a fundamental challenge in aging research and medical sciences. The neutrophil-to-lymphocyte ratio (NLR) is a readily measurable indicator of immune balance that reflects the interplay between innate immune activation and adaptive immune suppression. **Methods**: This study examined NLR values in 204 physically healthy residents (98 men and 106 women) stratified into four lifespan categories based on death certificates. Page’s test and ordinal regression (Cumulative Link Model) were used to assess trends with longevity. **Results**: In men, a downward trend in NLR values was observed. In women, a significant age-related decline in NLR was identified, with longer-lived individuals showing notably lower NLR values compared to their shorter-lived counterparts. The findings suggest that lower NLR is associated with longer survival, particularly in older women, reflecting superior immune regulation and reduced systemic inflammation. Conversely, elevated NLR may indicate immune dysfunction and heightened inflammatory burden. **Conclusions**: The results of this study complement existing findings, reinforcing the critical importance of immune balance in supporting healthy aging and longevity. These findings also underscore the potential of NLR as a robust biomarker for evaluating immune function and anticipating resilience to age-related decline, offering a practical tool for assessing immune health in the aging population.

## 1. Introduction

Human aging is a highly complex and multifaceted process that increases vulnerability to a wide spectrum of age-related pathologies, including cardiovascular diseases (CVD), cancer, type 2 diabetes mellitus (T2DM), pulmonary dysfunctions, autoimmune disorders, neurodegenerative disorders, as well as geriatric syndromes (e.g., frailty, chronic fatigue, gait disorders, and falls), but it also induces significant changes in the immune system, leading to immunosenescence [1,2]. This decline in immune function heightens the risk of infections, promotes chronic low-grade systemic inflammation, and diminishes the ability of effective immune responses in older people [3]. It has been established that chronic systemic inflammation plays a critical role in the onset and progression of numerous age-related diseases [4,5,6,7].

Among the biomarkers used to assess chronic systemic inflammation, the neutrophil-to-lymphocyte ratio (NLR) has emerged as a promising and easily accessible biomarker of immune dysregulation in aging. NLR, which is a simple and accessible metric derived from neutrophil and lymphocyte counts, provides a dynamic view of the immune landscape, encapsulating innate immune activation and adaptive immune suppression. This simple and cost-effective biomarker has recently emerged as a valuable tool for evaluating systemic inflammation and immune dysregulation in the context of aging. Furthermore, it has shown promise in predicting clinical outcomes, including physiological resilience and long-term survival [7,8,9,10].

The association between elevated NLR and adverse health outcomes is well-established in the literature. Elevated NLR has been linked to cardiovascular disease [11,12], acute stroke [13], and various cancers, including breast, lung, colorectal, pancreatic, prostatic, esophageal, and gastric malignancies [14,15,16,17,18,19,20,21,22]. It is also associated with atherosclerosis [23], severe trauma [24], infections [25], sepsis [26], acute pancreatitis [27,28], appendicitis [29], and all-cause mortality [30,31]. Furthermore, elevated NLR levels may have significant implications for Alzheimer’s disease [32], as higher NLR values are closely associated with an increased risk of dementia and heightened chronic systemic inflammation, which has been shown to exacerbate β-amyloid (Aβ) accumulation and neurofibrillary tangles (NFTs), further promoting neuroinflammation and neurodegeneration in elderly patients [33]. Nevertheless, previous studies have not consistently demonstrated significant associations between NLR and mortality due to cancer, stroke, or T2DM [34]. Moreover, the role of NLR as an independent predictor of long-term survival in older adults remains underexplored.

Beyond disease prediction, elevated NLR may reflect declining physiological resilience to stressors in older adults, as this indicator is increasingly recognized as a trans-prognostic biomarker of chronic inflammation and physiological stress [35,36]. As immune function deteriorates with advancing age, NLR increases, making it a potential biomarker for monitoring immunosenescence and inflammatory burden [35]. Furthermore, NLR increases with advancing age even in healthy populations [37], although these changes tend to be subtle. This increase in NLR might be associated with a higher risk of age-related morbidity and mortality, all the more so because NLR correlates positively with body mass index (BMI) and both systolic and diastolic blood pressure [37]. Given the global rise in aging populations, NLR is a readily accessible marker of systemic inflammation, which is linked to metabolic health [38,39]. Elevated NLR is associated with insulin resistance, which is a key driver of T2DM and CVD [40]. As elevated NLR might signal impaired glucose metabolism, some studies suggest that NLR is a valuable adjunct to plasma glycated hemoglobin (HbA1c) for assessing glycemic control and inflammatory status in patients with T2DM [41].

It is important to note that older adults in mental health care settings often face unique challenges, including cognitive impairments, comorbidities, and increased vulnerability to physical and psychological stressors. Altogether, these factors may exacerbate systemic inflammation and thus increase the pace of aging. However, research on NLR as a predictor of long-term survival in this population remains limited. Preliminary evidence suggests that individuals with lower NLR values live longer than those with higher NLR values.

The current study addresses this gap in the literature by analyzing historical cohort data to examine the relationship between long-term NLR trends and longevity in physically healthy older adults. The research also aims to clarify the potential of NLR as a prognostic biomarker, advancing our understanding of inflammatory biomarkers in predicting health outcomes and survival in older adults residing in mental health facilities.

## 2. Materials and Methods

### 2.1. Study Design and Population

This study was conducted in accordance with the principles of the Declaration of Helsinki. It utilized archival clinical data from physical examinations performed between 1958 and 2000 at the Mental Health Care Center in the vicinity of Zielona Góra, Poland. The research was approved by the institutional review board in 2007. To ensure data confidentiality, all medical records were fully anonymized, resulting in a comprehensive database that integrated both longitudinal and cross-sectional data. The study population consisted of older adults born between 1913 and 1930, all of whom had mild mental disorders and were residents of the medical facility, where they were continuously monitored by medical staff.

Participants were eligible for inclusion if they were residents of the center, had no diagnosed diseases during the study period, and were not receiving pharmacological treatment for either somatic or mental disorders. Individuals were excluded if they had severe mental disorders requiring treatment at the Mental Hospital or if they had been diagnosed with a serious disease (e.g., cancer, stroke, myocardial infarction etc.).

The longitudinal cohort comprised 68 men and 74 women, who were monitored continuously from ages 45 to 70 years. The cross-sectional cohort consisted of 204 individuals, including 98 men and 106 women, who were assessed during periodic clinical examinations at multiple intervals. Participants in both cohorts underwent standardized clinical evaluations conducted by trained medical personnel under consistent conditions at the same medical institution throughout the period under investigation.

Individuals in the cross-sectional cohort were categorized into four lifespan groups based on death certificate records: (1) short lifespan: 15 men (aged 50–58 years, mean age 53 years) and 12 women (aged 50–58 years, mean age 53 years), (2) medium lifespan: 26 men (aged 58–65 years, mean age 63 years) and 30 women (aged 58–65 years, mean age 63 years), (3) long lifespan: 42 men (aged 65–72 years, mean age 68 years) and 40 women (aged 65–72 years, mean age 68 years), and (4) very long lifespan: 15 men and 24 women, all aged 76 years or older.

Hematological data were extracted from medical records. Each participant underwent multiple clinical evaluations, yielding approximately 300 measurements per individual in the longitudinal dataset and dozens in the cross-sectional dataset, which ensured statistical robustness. All measurements were conducted systematically by trained medical personnel under standardized conditions in the medical institution. Blood samples were analyzed in the same hospital laboratory using uniform methodologies to ensure consistency. Given the unavailability of automated cell counters in Poland during the study period, blood cell counts were performed manually using a Bürker chamber and Giemsa-stained blood. This classical approach, which is recognized as a reference standard, ensured high accuracy, particularly in detecting anomalies.

### 2.2. Statistical Methods

Repeated measurements for each participant were aggregated to calculate reliable estimates of central tendency and variability, including arithmetic means, medians, percentiles, standard errors (SEs), and standard deviations (SDs). This approach minimized variability and enhanced the reliability of the findings. Descriptive statistics, such as arithmetic means, medians, and percentiles, were computed for these data.

Data normality was assessed with the Shapiro–Wilk test. The significance level was set at 0.05. Page’s trend test was employed to evaluate age-related trends in NLR values. The null hypothesis states that the measures of central tendency are equal across all analyzed groups. The alternative hypothesis posits that for the measures of central tendency in *n* studied groups—*θ*_1_, *θ*_2_, *θ*_3_, …, *θ_n_*—the following sequence of inequalities holds: *θ*_1_ ≤ *θ*_2_ ≤ *θ*_3_ ≤ … ≤ *θ_n_*, with at least one strict inequality. This indicates an upward trend in central tendency measures. In this analysis, this means that the median values of the analyzed variables increase across consecutive age groups: 45, 50, 55, 60, 65, and 70 years.

Ordinal regression was performed using the Cumulative Link Model (CLM), which incorporates covariates and offers a robust framework for modeling ordinal outcomes. All statistical analyses were conducted using R software (R Foundation for Statistical Computing, Vienna, Austria).

## 3. Results

NLR did not follow a normal distribution (Shapiro–Wilk test, *p* < 0.05). The key findings of the present study, including the statistical analysis of NLR values in the study population, are summarized in Table 1.

**Figure 1:**
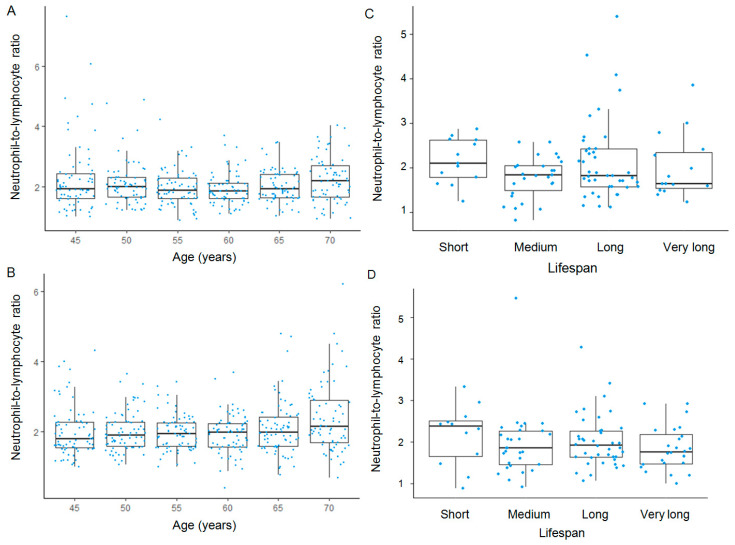
Age-related and survival-associated trends in NLR. Longitudinal trends in NLR with age are shown for men (**A**) and women (**B**), while cross-sectional survival-associated trends in NLR are depicted for men (**C**) and women (**D**). In the box-and-whisker plots, the bold line within each box represents the median, with the lower and upper edges indicating the first and third quartiles, respectively. Whiskers extend to the most extreme values within 1.5 times the interquartile range (IQR), and data points beyond this range are plotted as outliers.

In men, a trend of decreasing NLR with age was observed. Specifically, the highest median NLR was found in the short-lived participants, whereas the lowest median was observed in the long-lived individuals (Figure 1, Table 1). However, Page’s test did not reveal a statistically significant age-related trend (test statistic = 4944.5, *p* = 0.796).

A similar pattern was observed in women, where NLR values decreased with age. In contrast to the male cohort, the age-related decline in NLR was statistically significant (test statistic = 5616.5; *p* = 0.004). Furthermore, longer-lived women had significantly lower NLR values compared to their shorter-lived counterparts (Figure 1, Table 1).

The association between the predictor and the ordinal outcome was assessed using CLM, stratified by sex, with the results summarized in Table 2. In men, the analysis yielded an estimated coefficient of −0.187 (Standard Error = 0.125), corresponding to a *z*-value of −1.493 and a *p*-value of 0.135. The odds ratio (OR) was 0.829 (95% CI: 0.589–1.015). Similarly, in women, the estimate was −0.249 (Standard Error = 0.196), z-value = −1.271, *p*-value = 0.204, with an OR of 0.779 (95% CI: 0.525–1.153).

## 4. Discussion

This study builds on prior research examining trends in WBC, health outcomes, and longevity among older adults residing in the same mental health center [42,43,44,45,46,47], offering novel insights into the relationship between NLR and long-term survival. Our findings suggest that longer-lived individuals, particularly women, tended to have lower NLR levels compared to their shorter-lived counterparts. This likely reflects better-regulated immune and inflammatory responses, which are critical for resilience to chronic diseases and frailty in later life. By contrast, elevated NLR is linked to systemic inflammation, immune dysfunction, and increased mortality [48,49,50,51,52,53,54]. Although the direction of the associations suggested a potential inverse relationship between the predictor and the odds of experiencing higher outcome categories in both sexes, these findings did not achieve statistical significance. The estimated reduction in odds was 17.1% in men and 22.1% in women. On balance, these results may reflect a modest biological effect that the current sample size was underpowered to detect. Therefore, future studies with larger cohorts or meta-analytic approaches will be needed to clarify whether this finding represents a clinically meaningful relationship or merely statistical fluctuation.

It is well known that women generally display stronger immune responses than men, driven by estrogen and X chromosome-linked genes that enhance immune activity but also increase vulnerability to autoimmune diseases and systemic inflammation [42,55,56]. However, the postmenopausal decline in estrogen eliminates its anti-inflammatory effects, amplifying immune dysregulation and inflammaging. Men, on the other hand, experience a slower immune decline and more gradual hormonal changes with advancing age, resulting in less abrupt alterations in NLR.

An elevated NLR reflects an imbalance between an overactive innate immune system and a declining adaptive immune system. Neutrophils, central to the innate immune response, increase during systemic inflammation and stress, while lymphocytes, essential for adaptive immunity, decline with age due to thymic involution, reduced T-cell diversity, and impaired immune function. Chronic systemic inflammation, reflected in elevated NLR, drives the development of multiple age-related diseases, including metabolic dysfunction, cancer, and cardiovascular disease [4,5,7,56]. Neutrophils produce reactive oxygen species (ROS) during inflammation [53,57], and chronic ROS production promotes molecular damage to DNA, lipids, and proteins. This triggers a vicious cycle of oxidative damage, chronic inflammation, and cellular dysfunction, including mitochondrial dysfunction, accelerating biological aging and increasing susceptibility to CVD, cancer, and other age-related pathologies [1,5,58].

In long-lived individuals, lower NLR values might indicate a well-regulated immune response, whereas higher NLR values in shorter-lived individuals suggest systemic inflammation and immune dysregulation [34,36,37,48,49,50,51]. The clinical relevance of NLR as a biomarker spans multiple domains of age-related pathologies. In CVD, the leading cause of death worldwide, elevated NLR has been associated with myocardial infarction, stroke, and adverse outcomes in chronic heart failure [59,60].

**Figure 2:**
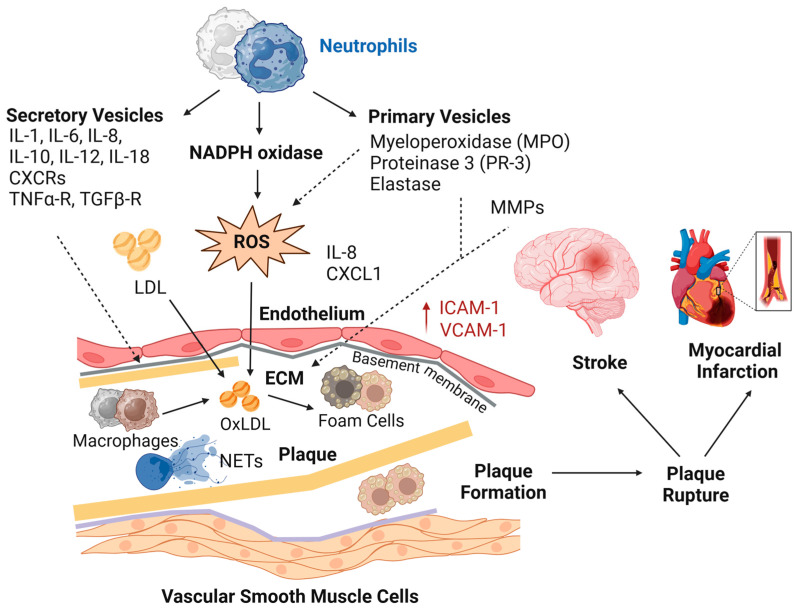
Neutrophils contribute to atherosclerosis by promoting monocyte recruitment via the chemokines interleukin-8 (IL-8) and CXCL1, while their interactions with platelets amplify vascular inflammation. Activated neutrophils generate reactive oxygen species (ROS), exacerbating oxidative stress and promoting the oxidation of low-density lipoproteins (OxLDL). The accumulation of OxLDL fuels foam cell formation, accelerating plaque development and destabilization, thereby increasing the risk of atherothrombosis and cardiovascular events. CXCRs = CXC chemokine receptors; ECM = extracellular matrix; ICAM-1 = intercellular adhesion molecule-1; ILs = interleukins; MMPs = matrix metalloproteinases; NADPH oxidase = nicotinamide adenine dinucleotide phosphate oxidase; NETs = neutrophil extracellular traps; TNFα-R = tumor necrosis factor alpha receptor; TGFβ-R = transforming growth factor beta receptor; VCAM-1 = vascular cell adhesion molecule-1.

Neutrophils play a crucial role in the initiation, progression, and destabilization of atherosclerotic plaques through the release of cytokines and ROS, proteolytic enzymes, and neutrophil extracellular traps (NETs) [52,53,60,61,62,63]. Upon recruitment to inflamed endothelium via chemokines such as CXCL1 and CXCL8, neutrophils exacerbate vascular inflammation by releasing pro-inflammatory cytokines, including IL-1β, IL-8, and tumor necrosis factor-α (TNF-α), which amplify endothelial dysfunction and promote monocyte adhesion. Neutrophil-derived ROS, primarily generated by nicotinamide adenine dinucleotide phosphate (NADPH) oxidase (NOX2) and myeloperoxidase (MPO), contribute to oxidative stress, endothelial damage, and the oxidation of low-density lipoproteins (OxLDL), which drive foam cell formation and atherogenesis [53,64]. Moreover, neutrophil elastase (NE) and matrix metalloproteinases (MMPs) degrade extracellular matrix (ECM) components, weakening the fibrous cap and increasing the risk of plaque rupture, which can lead to cardiovascular events such as stroke and myocardial infarction (Figure 2).

Neutrophil-derived NETs further promote intraplaque inflammation, enhance thrombus formation, and activate platelets, leading to a thromboinflammatory cascade [63]. These processes culminate in acute cardiovascular events, including stroke and myocardial infarction. Pro-inflammatory cytokines produced by neutrophils, including IL-6 and TNF-α, can disrupt insulin signaling by activating stress pathways such as c-Jun NH(2)-terminal kinase (JNK) and NF-κB, leading to insulin resistance and chronic metabolic stress. This metabolic dysfunction contributes to inflammaging, mitochondrial stress, and cellular senescence, all of which are strongly associated with accelerated aging and reduced health span and lifespan [1,4,5,7,30,37,42,43,58]. Furthermore, lymphopenia may reflect systemic stress and immune dysregulation, and the combination of chronic inflammation and lymphocyte depletion can further exacerbate vascular dysfunction, increasing the risk of CVD and mortality [30,42,65,66,67].

In clinical medicine, NLR has emerged as a robust and accessible biomarker of systemic inflammation and a harbinger of poor prognosis [6,8,41,49,54]. Defined as the quotient of the absolute neutrophil count to the absolute lymphocyte count, typically derived from peripheral blood samples, this index not only reflects the dynamic balance between the innate and adaptive immune responses but also serves as a practical tool in both diagnostic and prognostic evaluations. In selected instances, analogous indices can be derived from histopathological examinations by quantifying inflammatory cell infiltrates within tissue specimens or neoplastic lesions, thereby offering localized insights into inflammatory processes. The clinical relevance of NLR is particularly evident in inflammatory diseases characterized by granulomatous immune responses, including vasculitides, inflammatory bowel disease (IBD), and autoimmune disorders. In parallel, the lymphocyte-to-monocyte ratio (LMR) has been increasingly explored as a complementary biomarker in chronic inflammatory conditions such as tuberculosis and certain malignancies, further expanding the spectrum of immune-based indices used in clinical and prognostic assessments [68,69,70].

Evidence from experimental and observational studies has substantiated the prognostic value of NLR across a spectrum of clinical settings. For example, in stroke, elevated NLR levels have been consistently associated with increased stroke severity, poorer functional outcomes, and higher mortality rates [12,13]. Similarly, in cardiology, a high NLR has been linked to adverse procedural outcomes and increased mortality, underscoring its potential as a prognostic biomarker in cardiovascular risk stratification [11,12].

Given the wide-ranging implications of NLR across these diverse domains of health, future research will need to focus on elucidating the precise molecular mechanisms linking NLR to disease pathogenesis, particularly in the context of aging, metabolic dysfunction, and cardiovascular health [12,38,52,53,54,56,57,58]. In the context of CVD, for example, neutrophils, as key drivers of inflammation, are involved in the pathogenesis of atherosclerosis and its complications, including myocardial infarction and stroke [11,12,13]. Neutrophils, which increase during systemic inflammation, contribute to endothelial injury through the release of proteolytic enzymes and ROS. These cells can also promote plaque destabilization and rupture in atherosclerosis [52,61], in part by forming NETs, which facilitate thrombosis [63]. Once ruptured, these plaques can trigger the formation of blood clots, which can obstruct blood flow and result in myocardial infarction or stroke, as illustrated in Figure 2. Moreover, elevated NLR is associated with the activation of key inflammatory mediators, such as IL-6, C-reactive protein (CRP), and TNF-α, which can amplify systemic inflammation and promote the pathogenesis of chronic diseases. In particular, IL-6 is an interleukin that acts as both a pro-inflammatory and an anti-inflammatory cytokine, which is secreted by macrophages and T cells. The broad implications of NLR as a biomarker of systemic inflammation extend to aging and age-related diseases, where chronic low-grade systemic inflammation, which is often referred to as inflammaging, plays a central role in accelerating aging and age-related pathologies, including CVD, T2DM, and cancer [4,7,30,33,67,70,71,72].

**Figure 3:**
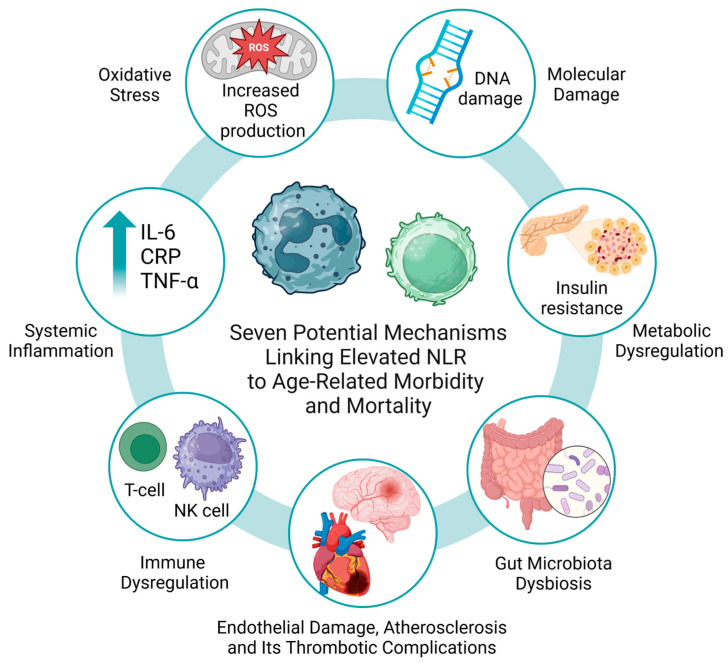
Seven possible mechanisms linking elevated NLR to age-related morbidity and mortality.

Oncological research further supports the negative prognostic implications of elevated NLR, as higher NLR has been consistently associated with poorer overall survival across various solid tumors, as demonstrated by numerous studies, including several meta-analyses [73,74]. Elevated NLR serves as a robust prognostic biomarker of tumor progression across multiple cancers, including lung, breast, gastric, colorectal, and pancreatic malignancies [14,15,16,17,18,19,20,21,22]. For example, in malignancies of the gastrointestinal tract, elevated NLR has been correlated with aggressive tumor biology, reduced response to therapy, and diminished overall survival [18,22]. Neutrophils can promote tumor progression and metastasis through the release of growth factors, pro-angiogenic factors, and matrix-degrading enzymes that remodel ECM and facilitate invasion. Conversely, lymphopenia can impair immunosurveillance by reducing cytotoxic T-cell and natural killer (NK) cell activity, thereby diminishing the host’s ability to eliminate malignant cells. These mechanistic insights highlight the prognostic relevance of the NLR and support its potential utility in guiding therapeutic strategies (Figure 3).

Furthermore, emerging evidence links gut microbiota disturbances (dysbiosis) with chronic systemic inflammation [5,58]. The gut-derived cytokines might enhance neutrophilic activity and suppress lymphocyte function. The intricate relationship between gut microbiota and systemic inflammation has gained significant attention in aging research [5]. Dysbiosis refers to an imbalance in the composition and functionality of the gut microbiome, with substantial implications for health. It plays a critical role not only in the aging process, as one of its hallmarks, but also in the development of age-related dysfunctions and several diseases [5,58]. This dysregulation of the gut microbiome profoundly impacts immune system modulation. It is hypothesized that the gut microbiota plays a crucial role in shaping immune responses and maintaining homeostasis. In dysbiosis, a depletion of beneficial microbes coupled with an overgrowth of pathogenic bacteria can trigger the release of pro-inflammatory cytokines (e.g., IL-6, TNF-α, and CRP), leading to increased systemic inflammation [4,7,30].

**Figure 4:**
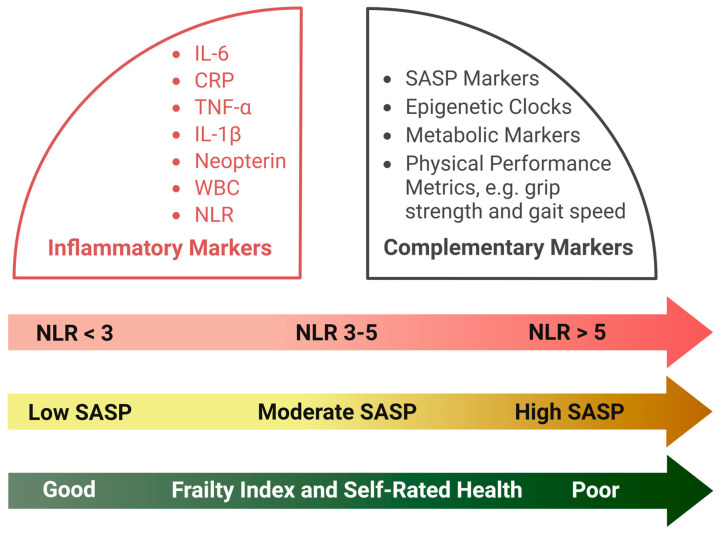
A comprehensive biomarker panel comprising inflammatory markers and emerging indicators of aging and longevity.

However, the utility of NLR as a biomarker extends beyond its ability to reflect immune and inflammatory states, as high NLR levels also correlate with markers of the senescence-associated secretory phenotype (SASP), including interleukin-6 (IL-6) and TNF-α, which drive chronic inflammation, tissue damage, and immune dysregulation. Combining NLR with SASP biomarkers, metabolic indicators, physical performance metrics, which are strong predictors of age-related morbidity and mortality [30,75,76,77,78,79,80,81,82] will provide a comprehensive framework for assessing overall health and identifying individuals at risk of frailty, functional decline, and mortality. Furthermore, integrating these measures with emerging tools, including epigenetic clocks such as GrimAge, Hannum, Horvath, and PhenoAge [82], can help in developing multidimensional age metrics and highly accurate survival models (Figure 4). For instance, elevated NLR combined with accelerated GrimAge scores and poor physical performance metrics, including grip strength [76,77] and gait speed [78], as well as frailty index and self-rated health [80,81] may indicate accelerated aging, while lower NLR values alongside favorable biomarker profiles might reflect enhanced physical resilience and healthy aging.

Interestingly, recent studies have reported an association between elevated NLR values and an increased risk of delirium, particularly in older adults with comorbidities [83]. Delirium, especially the hypoactive subtype, is a strong and independent predictor of death and the need for intensive care, yet it remains underrepresented in mortality prediction models. Older adults with cognitive impairment, dementia, or a high comorbidity burden are particularly vulnerable. Di Giorgio et al. (2022) demonstrated that each ten-unit increase in NLR was associated with a 45% increase in delirium risk [83]. These findings point to the critical role of inflammation in the pathogenesis of delirium, which can be mediated by neuroinflammatory mechanisms such as microglial activation and blood–brain barrier disruption. Given the prognostic significance of delirium and inflammatory markers, early identification of high-risk patients, particularly those with cognitive disorders and comorbidities, may be essential for improving outcomes.

In summary, given the central role of chronic systemic inflammation in biological aging and its associated pathologies, our study sought to examine the relationship between NLR and longevity in physically healthy individuals over an extended period. Our findings demonstrated an age-related decline in NLR in older women, with those who lived longer tending to exhibit lower NLR values compared to their peers with shorter lifespans. This pattern suggests that a lower NLR reflects superior immune regulation and diminished systemic inflammation. Conversely, elevated NLR might be indicative of immune dysregulation and heightened inflammatory burden, potentially accelerating the aging process.

This study has limitations that need to be acknowledged. First, the observational and retrospective nature of our analysis, which relied on historical data, renders the findings susceptible to both recognized and unrecognized confounding factors, cautioning against broad generalizations. Second, our sample was drawn from a relatively specific population of individuals residing in a mental health center. Third, while the inflammation parameters under scrutiny are known to fluctuate rapidly—due to dynamic physiological processes such as endothelial adhesion or cellular recruitment—the aggregation of repeated measurements over five-year intervals helped mitigate the influence of these transient effects.

On the other hand, our results contribute to a growing body of evidence positioning NLR as a promising biomarker of survival. This study not only reinforces the clinical relevance of NLR in evaluating systemic inflammation but also opens avenues for future research. Understanding the molecular and cellular mechanisms underlying inflammaging could lead to novel interventions aimed at promoting healthier, longer lives, which is the ultimate goal of geroscience. Future research should integrate multidisciplinary approaches to gain deeper insights into the complex interplay between immune function, inflammatory processes, and health outcomes in aging populations.

## Figures and Tables

**Table 1:** NLR values and relevant descriptive statistics for men and women across the four survival categories.

Lifespan Category	Men						Women					
	Min	Q_1_	Median	Q_3_	Max	Mean (± SD)	Min	Q_1_	Median	Q_3_	Max	Mean (± SD)
Short	1.3	1.8	2.3	2.7	2.9	2.2 (0.7)	0.8	1.6	2.4	2.5	3.3	2.2 (0.7)
Medium	0.8	1.5	1.8	2.0	2.6	1.8 (0.5)	0.9	1.5	1.9	2.3	5.3	2.1 (0.8)
Long	1.1	1.6	1.8	2.4	5.4	2.1 (1.0)	1.1	1.6	1.9	2.3	4.3	2.0 (0.6)
Very long	1.2	1.5	1.6	2.3	3.9	2.0 (0.7)	1.0	1.5	1.7	2.2	2.9	1.8 (0.5)

**Table 2:** Cumulative Link Model (CLM) outcomes in both sexes.

Sex	Estimate	Standard Error	z-Value	Pr (>|z|)	Odds Ratio	2.5%	97.5%
Men	−0.187	0.125	−1.493	0.135	0.829	0.589	1.015
Women	−0.249	0.196	−1.271	0.204	0.779	0.525	1.153

## Data Availability

The data presented in this study are available on request from the corresponding author, as these data are not publicly available due to privacy and ethical restrictions.
